# Protective Effects of Ambient Ozone on Incidence and Outcomes of Ischemic Stroke in Changzhou, China: A Time-Series Study

**DOI:** 10.3390/ijerph14121610

**Published:** 2017-12-20

**Authors:** Yongquan Yu, Huibin Dong, Shen Yao, Minghui Ji, Xingjuan Yao, Zhan Zhang

**Affiliations:** 1 Department of Occupational Medicine and Environmental Health, School of Public Health, Nanjing Medical University, 101 Longmian Avenue, Nanjing 211166, China; yyqaion@sina.com (Y.Y.); 13814128712@163.com (S.Y.); jiminghui77@sina.com (M.J.); 2 Department of Chronic Disease Control and Prevention, Changzhou Center for Disease Control and Prevention, 203 Taishan Road, Changzhou 213022, China; huibind@126.com (H.D.); myaoxj@163.com (X.Y.); 3 Department of Hygiene Analysis and Detection, School of Public Health, Nanjing Medical University, 101 Longmian Avenue, Nanjing 211166, China

**Keywords:** ozone, ischemic stroke, time-series study, generalized additive model

## Abstract

The potential beneficial effect of ozone (O_3_) on stroke had been identified experimentally and clinically, but these effects remain controversial in population-based studies. This study aimed to explore the epidemiological association between O_3_ and risk of ischemic stroke. Ischemic stroke related health data and air pollution data were obtained from the Center for Disease Control and Prevention and Environmental Monitoring Center in Changzhou between 2015 and 2016, respectively. The associations between the short-term exposure to O_3_ and daily ischemic stroke onsets and deaths were examined based on time-series generalized additive Poisson model. During the study period, daily ischemic stroke onsets and deaths decreased 0.340% (95% confidence interval (CI) −0.559% to −0.120%) and 0.697% (95% CI −1.103% to −0.290%) with an interquartile range (IQR) (41.1 µg/m^3^) increase in levels of ambient O_3_, respectively. The protective effects of O_3_ were more significant in men and elders and in the cool season than those in women and young people and in the warm season, respectively. The negative association was independent of PM_2.5_, PM_10_, SO_2_, NO_2_ or CO exposure. Acute O_3_ exposure was associated with decreased risk of ischemic stroke. These findings will help provide new insights into the relationship between ischemic stroke and ambient O_3_ concentrations.

## 1. Introduction

Stroke is the leading cause of death and disability-adjusted life years worldwide; approximately 16.9 million new stroke cases and 5.9 million stroke related deaths occurred in 2010 [1,2,3]. Ischemic stroke occurs when a blood vessel carrying blood to the brain is blocked by arterial thrombosis or embolism [4]. It is the major subtype of stroke (accounting for approximately 80% of all strokes) and can result in neurologic cell death and dysfunction [4,5]. Due to the enormous disease burden of ischemic stroke, numerous studies were conducted to detect the modifiable risk factors for ischemic stroke, including potentially deleterious air pollutants [6,7]. A cohort study in Hong Kong found that exposure to fine particulate matter (PM) with aerodynamic diameter less than 2.5 μm (PM_2.5_) were associated with higher risk of ischemic stroke [8]. An interquartile range (IQR) increase in daily average levels of PM_10_ was also positively associated with a 0.8% increase in ischemic stroke admissions [9]. Moreover, short-term increments in gaseous pollutants such as nitrogen dioxide (NO_2_), sulfur dioxide (SO_2_) and carbon monoxide (CO) were found to be associated with higher ischemic stroke risk [10,11]. However, only a few researches have observed the cerebrovascular effect of ozone (O_3_) and the results were controversial. Few of them found statistical positive associations between risk of ischemic stroke and daily O_3_ concentrations [12], while others found no significant associations [13,14]. Thus, it is an urgent need to document the relationship between ambient O_3_ levels and risk for ischemic stroke.

Ground-level O_3_ is a pale blue secondary pollutant, which is created by the action of ultraviolet rays on precursors such as combustion of fossil fuels and methane [15]. However, under certain circumstance, O_3_ may also serve as a ‘protector’ against cerebral damage. O_3_ major auto-hemo-therapy has been used in the treatment of ischemic disorders [16]. Oxygen-ozone gaseous mixture appeared to be effective in reverting damage of brain tissues [17]. It also can exert neuroprotective effects by regulating inflammatory response, improving cerebrovascular rheology and strengthening antioxidant in hypoxic brains [18].

In the current study, a time-series analysis was conducted to examine the associations between the short-term increment in ambient O_3_ concentrations and daily ischemic stroke onsets and deaths in Changzhou. Two-pollutant and multi-pollutant models were used to control the confounding pollutants. Sub-group analysis was conducted to explore the effect modification and screen the sensitive subpopulations. Acute O_3_ exposure was associated with decreased risk of ischemic stroke. This study will help provide new insights into the correlation between ischemic stroke and ambient O_3_ exposure.

## 2. Materials and Methods

### 2.1. Data Collection

Data on daily onsets and deaths for ischemic stroke were collected from cardio-cerebrovascular disease reporting system of Changzhou between 9 January 2015 and 31 December 2016. The diagnosis of ischemic stroke was on the basis of The International Classification of Diseases, Revision 10 (ICD-10) codes for ischemic stroke (I63). There were approximately 45.4 ischemic stroke cases (32,840 in total) and 5.6 ischemic stroke deaths (4028 in total) identified each day. The daily ischemic stroke counts and deaths were further stratified into groups by sex, age (young people <65 years and elders ≥65 years) and season (warm season as 1 May to 31 October and cold season as 1 November to 30 April). For the daily ischemic stroke counts, the average number in male and elder was 23.7 and 36.9, respectively. For daily ischemic stroke deaths, the average number in male and elder was 2.7 and 5.1, respectively.

Daily mean levels for ambient O_3_ (90.7 µg/m^3^, 8 h max) and other air pollutants such as PM_2.5_ (51.8 µg/m^3^), PM_10_ (85.3 µg/m^3^), NO_2_ (22.3 µg/m^3^), SO_2_ (38.7 µg/m^3^) and CO (1 mg/m^3^) were collected from the database of Changzhou Environmental Monitoring Center (EMC) from 9 January 2015 to 31 December 2016. The average daily temperature and relative humidity was 17.0 °C and 75%, respectively. A total of six state-controlled, two province-controlled and two city-controlled air quality monitoring stations were established to comprehensively record the variation of air pollutants levels. The air pollution concentrations used in this study were averaged from all the stations as they all represented the urban background air pollution levels. Moreover, the fixed-site air pollution data might be a good proxy for personal exposure as all the ischemic stroke patients and deaths were resided less than 40 km from the nearest station [19]. The present study was approved by the Institutional Review Board of Changzhou Center for Disease Control and Prevention, and all procedures were in accordance with prevailing ethical principles (ethnical code: 2016-01).

### 2.2. Statistical Analysis

Daily data for air pollutants levels and weather conditions were pooled together for the same time to match the daily ischemic stroke onsets and deaths. A longitudinal time-series design was conducted to evaluate the associations between the short-term expose to O_3_ and daily ischemic stroke onsets and deaths. Generalized additive model (GAM) with Poisson regression was used to calculate data and multivariable regression model was used to control the potential confounding factors:

$$\begin{array}{cc} \mathrm{Log}\left[E\left(T_{dn}\right)\right]= & \mathrm{intercept}+\beta_{1}O_{3\left(n-i\right)}+\beta_{2}DOW+ps\left(calender\;time,\;df=7\right)+ps\left(Temp_{n-i},\;df=5\right)\\ & \;+ps\left(Relatuve\;Humidiy_{n-i},\;df=5\right) \end{array}$$

where $E\left(T_{dn}\right)$ was the expected daily numbers of ischemic stroke onsets or deaths on day n; $O_{3\left(n-i\right)}$ was the daily mean levels of O_3_ on day n and i was the day lag; β was vector of the coefficients; *DOW* was the day of week; *ps* () meant penalized spline function; $Temp_{n-i}$ and $Relative\;Humidity_{n-i}$ meant average temperature and relative humidity on day n and i represented the day lag.

To detect the possible lagged effects that may occur, lag models were used with different lag days (lag 0, lag 1, lag 3 and lag 5). Furthermore, the moving average levels of O_3_ during 0–1 days (lag 0–1), 0–3 days (lag 0–3) and 0–5 days (lag 0–5) were detected to comprehensively evaluate the effects. Two-pollutant models by respectively adjusting PM_2.5_, PM_10_, NO_2_, SO_2_ and CO levels and multi-pollutant model by adding all the pollutants were conducted for the subsequent sensitivity analysis. Smoothing function was also used to graphically analyze the exposure-response association between daily O_3_ levels and log-relative risk of daily ischemic stroke onsets or deaths with a 5 df in single-pollutant model.

Statistical software R (version 3.2.3, R Foundation for Statistical Computing, Vienna, Austria) was used for data analysis and result output. The baseline data were presented as mean ± standard deviation (SD) for continuous variables. All tests were two-sided, and *p* < 0.05 was considered as statistically significant.

## 3. Results

**Figure 1:**
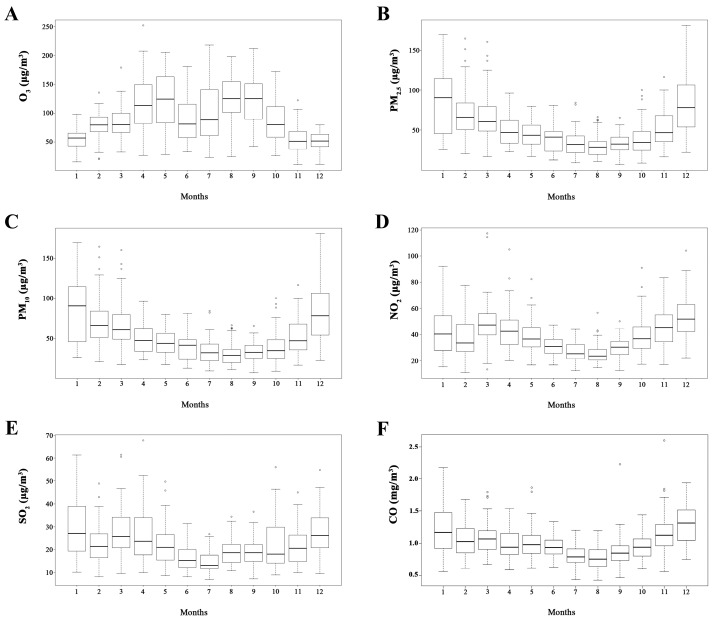
Monthly distribution patterns of ambient pollutants in Changzhou, 2015–2016. (**A**) O_3_ indicates ozone; (**B**) PM_2.5_, particulate matter that is ≤2.5 µm in diameter; (**C**) PM_10_, particulate matter that is ≤10 µm in diameter; (**D**) NO_2_, nitrogen dioxide; (**E**) SO_2_, sulfur dioxide; (**F**) CO, carbon monoxide.

The monthly distribution patterns of O_3_ and other ambient pollutants (PM_2.5_, PM_10_, SO_2_, NO_2_ and CO) were illustrated in Figure 1. The concentration of O_3_ was higher in warm seasons than in cool seasons (Figure 1A), whereas the concentrations of PM_2.5_ (Figure 1B), PM_10_ (Figure 1C), NO_2_ (Figure 1D), SO_2_ (Figure 1E), and CO (Figure 1F) were higher in cool seasons than in warm seasons.

**Figure 2:**
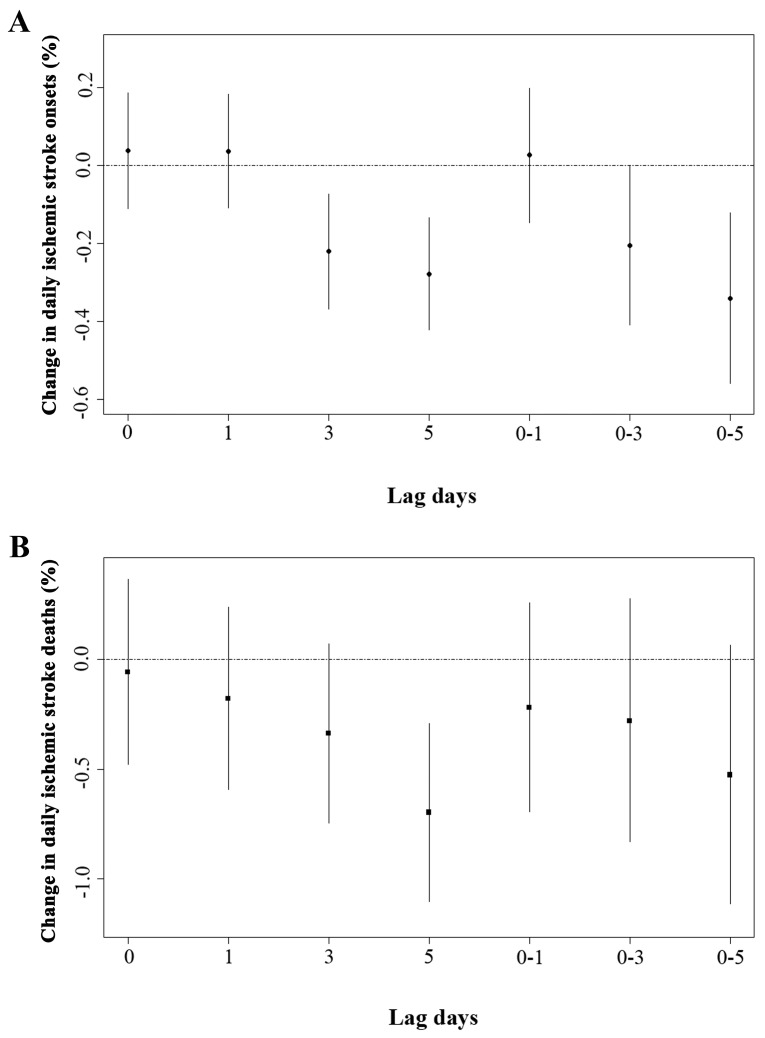
The percentage changes in daily ischemic stroke onsets (**A**) and deaths (**B**) with per interquartile range increment in O_3_ concentrations at different lag days in single-pollutant models in Changzhou, 2015–2016. The data was expressed as mean with 95% confidence interval. O_3_ indicates ozone.

The results of the single-pollutant model for daily ischemic stroke onsets and deaths were summarized in Figure 2. Statistical percent decreases (95% confidence interval (CI)) were observed in daily ischemic stroke onsets with an interquartile range (IQR) (41.1 µg/m^3^) increase in O_3_ concentrations for lag 3 (−0.220%, 95% CI −0.367% to −0.072%), lag 5 (−0.278%, 95% CI −0.421% to −0.134%) and lag 0–5 (−0.340%, 95% CI −0.559% to −0.120%) days and the association was more pronounced for lag 0–5 than lag 3 and lag 5 (Figure 2A). Therefore, lag 0–5 was used in the subsequent analysis. Negative association was also found between O_3_ levels and daily ischemic stroke deaths with a 5 day lag (lag 5). An IQR increment of O_3_ corresponded to 0.9% decrease of daily ischemic stroke deaths. No statistical association was observed at other lag days (Figure 2B).

Subgroup analyses modifying by age, gender and seasons were presented in Table 1. For daily ischemic stroke onsets, statistically negative associations were only observed among men and elders. In details, the percent decrease in daily ischemic stroke onsets with an IQR increase in O_3_ levels was 0.434% for men and 0.4% for elders. Moreover, the estimated effect of O_3_ was more significant in the cool season (−0.845%, 95% CI −1.361% to −0.326%) than in the warm season (−0.076%, 95% CI −0.398% to 0.246%). As for daily ischemic stroke deaths, similarly, statistically negative associations were also found among men (−1.040%, 95% CI −1.612% to −0.464%) and elders (−0.697%, 95% CI −1.120% to −0.273%). Moreover, the negative association in the cool season group (−1.006%, 95% CI −1.839% to −0.165%) was more pronounced than that in the warm season group (−0.714%, 95% CI −1.203% to −0.223%).

**Figure 3:**
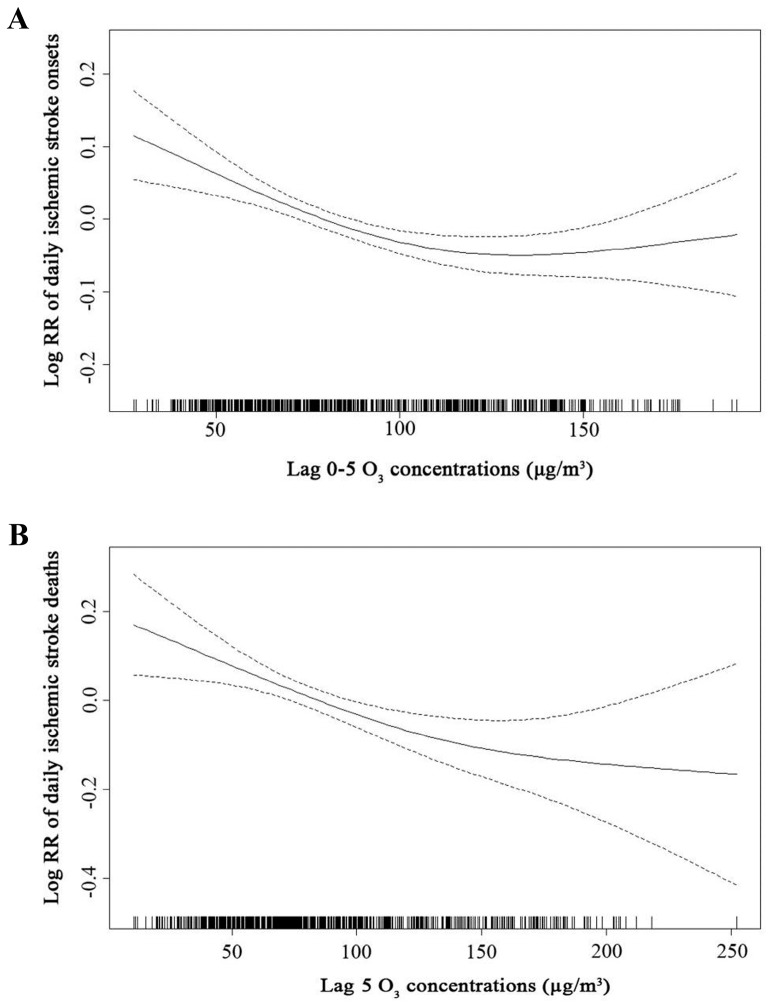
The exposure-response relationships of O_3_ levels with ischemic stroke onsets (**A**) and deaths (**B**) at selected lag days (lag 0–5 for ischemic stroke onsets and lag 5 for daily ischemic stroke deaths) in single-pollutant models in Changzhou, 2015–2016. The data was expressed as mean with 95% confidence interval. O_3_ indicates ozone.

**Figure 4:**
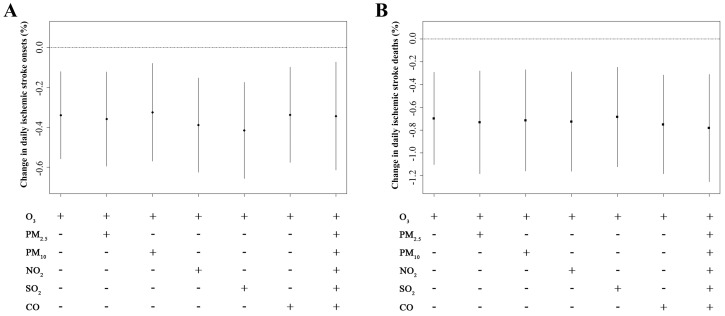
The percentage changes in daily ischemic stroke onsets (**A**) and deaths (**B**) with per interquartile range increment in O_3_ concentrations at selected lag days (lag 0–5 for ischemic stroke onsets and lag 5 for daily ischemic stroke deaths) in two-pollutant and multi-pollutant models in Changzhou, 2015–2016. The data was expressed as mean with 95% confidence interval. O_3_ indicates ozone; PM_2.5_, particulate matter that is ≤2.5 µm in diameter; PM_10_, particulate matter that is ≤10 µm in diameter; NO_2_, nitrogen dioxide; SO_2_, sulfur dioxide; CO, carbon monoxide.

The exposure-response relationships of O_3_ levels with risk of ischemic stroke onsets and deaths were performed in Figure 3. The curve for daily ischemic stroke deaths was approximately linear negative (Figure 3B), whereas the smoothing spline for daily ischemic stroke onsets was negatively linear and flat at higher O_3_ levels (Figure 3A). Moreover, sensitivity analysis was also conducted in Figure 4. In both two-pollutant and multi-pollutant models, the effect estimates of O_3_ were stable for both daily ischemic stroke onsets (lag 0–5) (Figure 4A) and daily ischemic stroke deaths (lag 5) (Figure 4B).

## 4. Discussion

In the present study, by recording 32,840 ischemic stroke counts and 4028 ischemic stroke-related deaths, negative associations between short-term ozone exposure and daily ischemic stroke onsets or deaths were identified in Changzhou from 9 January 2015 to 31 December 2016. The beneficial effects of O_3_ were more significant in men, elders (>65 years) and cool season. The exposure–response curve for ischemic stroke onsets was sharply linear negative at low O_3_ levels (<100 µg/m^3^), suggesting that the low environmental O_3_ exposure might be beneficial more against ischemic stroke onsets and deaths. Moreover, the O_3_ related neuroprotective effect was independent of PM_2.5_, PM_10_, SO_2_, NO_2_ or CO exposure. Our results might have implications in preventing and treating ischemic strokes.

Previous epidemiological and clinical studies have presented incontrovertible and deleterious effects of ambient air pollutants (such as PM_2.5_, NO_2_ and SO_2_) on the morbidity and mortality of ischemic stroke [9,20,21]. However, in terms of O_3_, few studies assessed its cerebrovascular effects and no consensus has been reached. Although no statistical significance, several studies have demonstrated a negative association between average O_3_ levels and risk of strokes, which was consistent with our results. The estimate odds ratio (OR) for the association between ischemic stroke hospitalizations and O_3_ exposure was 0.98 in South Carolina and the OR for the association between previous day O_3_ increments and odds of recurrent stroke was 0.97 in Nueces County, Texas [13,14]. Moreover, as shown in a multicenter cohort study, adjusted OR of ischemic stroke for one standard deviation (SD) increase in O_3_ was 0.96 and men was more sensitive to the protector role of O_3_ (OR = 0.76) [22].

In ischemic strokes, the cerebral blood supply is depleted due to the embolisms or thrombosis [23]. Then the concentrations of blood oxygen and the formation of high energy phosphate compounds are dramatically decreased, leading to the depolarization, inflammation and apoptosis of the brains [24,25]. It has been reported that O_3_ oxidative preconditioning could attenuate ischemia/reperfusion induced hepatic and renal damages in rats. The enhanced expressions of eNOS and iNOS induced by O_3_ could improve and reestablish the balance of cellular redox [26,27]. O_3_ therapy was found to have extensive clinical applications in improving hemorheological parameters and oxygen delivery in patients with peripheral occlusive arterial disease [28]. In addition, the neuroprotective concentration-response curve for O_3_ was shaped as ‘U’ with efficient level range of 80–120 µg/mL in rat models [17]. Thus, it is biologically plausible that within a selective concentration range, ambient O_3_ may exert neuroprotective effects against ischemic stroke onsets and deaths.

In this study, the protective effects of O3 on ischemic stroke onsets and deaths were found to be stronger in men than in women. Rather than vascular risk factors related differences between genders [29,30], the underlying reason for this observation might be the higher rate of ambient O_3_ exposure in men than in women due to longer time with outdoor working and laboring [31]. Thus, they are more likely to be exposed and affected by ambient O_3_. Moreover, the negative associations between air O_3_ levels and ischemic stroke onsets or deaths appeared to be more pronounced in elders than in young populations. Age is a nonmodifiable risk factor for ischemic stroke, and studies have indicated that ischemic tolerance is impaired in aged murine and human [32,33,34]. It has also been found that cerebral inflammation milieu is worse and the cerebrovascular hemodynamics is lower in elders than in young populations [35,36,37]. Therefore, elders are the plausibly sensitive subpopulations upon O_3_ exposure.

Season differences were also conducted in the current study. Because levels of air pollutants are variably depending on weather conditions, season is normally considered as a vital modifier in ambient air pollution related biological effects [38]. As a secondary pollutant, the formation of O_3_ requires nitrogen oxides, hydrocarbons and sunlight. Consequently, O_3_ levels are routinely higher in the warm season than in the cool season due to the increment of photochemistry (as shown in Figure 1). Furthermore, according to the concentration-response curves for both ischemic stroke onsets and deaths in this study, the negative associations were stronger at the O_3_ concentrations below the 100 µg/m^3^, a concentration range normally seen in cool season. Thus, it was reasonable that the protective effects for both ischemic stroke onsets and deaths were more significant in the cool season than in warm season.

Deleterious effects of air pollution on human cardio-cerebral vascular systems have been extensively reported. In a meta-analysis of 94 studies, an increment in levels of ambient pollutants was positively associated with increased admission for stroke or mortality stroke. The pooled relative risks were 1.011 for PM_2.5_, 1.002 for PM_10_, 1.015 for CO, 1.019 for SO_2_ and 1.014 for NO_2_, respectively [39]. And the underlying mechanism by which air pollution exert as a risk factor for ischemic stroke might be that PMs and gaseous air pollutants, such SO_2_ and NO_2_ could activate astrocytes, disrupt synaptic functions and enhance endothelial and inflammatory responses of the brains [40,41,42,43]. Thus, to obtain more reliable and precise results, the potential air pollutants confounders should be taken into considerate. As shown in the sensitivity analysis, the estimated percent changes in daily ischemic stroke onsets and deaths with per IQR increment in O_3_ levels were coincident and stable in both two-pollutant model and multi-pollutant model, suggesting that the neuroprotective effect of O_3_ was reliable and independent from other air pollutants.

Several weaknesses of this study should be noted. Although air pollution data were collected from ten fixed-site monitoring stations, they could not be representative enough of individual exposure. Thus, exposure fallacy may exist. Furthermore, due to the retrospective design of this study, detailed population data respecting cigarette smoking, type of indoor home heating, residential mobility and other potential risk factors for ischemic stroke such as high blood pressure and obesity were limited. In addition, the results presented in this study should be interpreted with cautions. As the relationship between O_3_ exposure and decreased ischemic stroke risk may not equal to the cause-effect association. Further epidemiological studies (such as cohort study) are needed to explore the O_3_ related long-term effects. The current study also possessed several strengths. By identifying a crowd of ischemic stroke onsets and deaths, this study provides sufficient statistical power to evaluate the O_3_ related effects. Moreover, the acute protective effect of O_3_ on ischemic stroke was comprehensively assessed and the results are reliable and valuable in this study.

## 5. Conclusions

In conclusion, the current population-based time-series study indicated that acute O_3_ exposure was associated with decreased risk of ischemic stroke. The neuroprotective effect was more robust in the cool season, and among women and elders.

## Figures and Tables

**Table 1:** Subgroup analyses of the associations between ambient O_3_ levels and daily ischemic stroke onsets and deaths modifying by age, gender and seasons.

Variables	Ischemic Stroke Onsets	Ischemic Stroke Deaths
Total	−0.340 (−0.559, −0.120) ^†^	−0.697 (−1.103, −0.290) ^‡^
Age ≤ 65	−0.098 (−0.585, 0.391)	−0.615 (−2.018, 0.809)
Age > 65	−0.400 (−0.643, −0.157) ^†^	−0.697 (−1.120, −0.273) ^†^
Men	−0.434 (−0.738, −0.13) ^†^	−1.040 (−1.612, −0.464) ^‡^
Women	−0.267 (−0.584, 0.051)	−0.387 (−0.960, 0.188)
Cold Season	−0.845 (−1.361, −0.326) ^†^	−1.006 (−1.839, −0.165) *
Warm Season	−0.076 (−0.398, 0.246)	−0.714 (−1.203, −0.223) ^†^

The data was expressed as mean with 95% confidence interval, * *p* < 0.05, ^†^ *p* < 0.01, ^‡^ *p* < 0.001.
